# Structural Insights into CB1 Receptor Biased Signaling

**DOI:** 10.3390/ijms20081837

**Published:** 2019-04-13

**Authors:** Rufaida Al-Zoubi, Paula Morales, Patricia H. Reggio

**Affiliations:** 1 Department of Medicinal Chemistry and Pharmacognosy, Faculty of Pharmacy, Jordan University of Science & Technology, P.O.BOX 3030, Irbid 22110, Jordan; rmalzoubi1@just.edu.jo; 2 Departamento de Química-Física Biológica, Instituto de Química Física Rocasolano (IQFR-CSIC), Serrano 119, 28006 Madrid, Spain; pmorales@iqfr.csic.es; 3 Chemistry and Biochemistry Department, UNC Greensboro, Greensboro, NC 27412, USA

**Keywords:** CB1 receptor, biased signaling, functional selectivity, cannabinoids

## Abstract

The endocannabinoid system has emerged as a promising target for the treatment of numerous diseases, including cancer, neurodegenerative disorders, and metabolic syndromes. Thus far, two cannabinoid receptors, CB1 and CB2, have been discovered, which are found predominantly in the central nervous system (CB1) or the immune system (CB2), among other organs and tissues. CB1 receptor ligands have been shown to induce a complex pattern of intracellular effects. The binding of a ligand induces distinct conformational changes in the receptor, which will eventually translate into distinct intracellular signaling pathways through coupling to specific intracellular effector proteins. These proteins can mediate receptor desensitization, trafficking, or signaling. Ligand specificity and selectivity, complex cellular components, and the concomitant expression of other proteins (which either regulate the CB1 receptor or are regulated by the CB1 receptor) will affect the therapeutic outcome of its targeting. With an increased interest in G protein-coupled receptors (GPCR) research, in-depth studies using mutations, biological assays, and spectroscopic techniques (such as NMR, EPR, MS, FRET, and X-ray crystallography), as well as computational modelling, have begun to reveal a set of concerted structural features in Class A GPCRs which relate to signaling pathways and the mechanisms of ligand-induced activation, deactivation, or activity modulation. This review will focus on the structural features of the CB1 receptor, mutations known to bias its signaling, and reported studies of CB1 receptor ligands to control its specific signaling.

## 1. Introduction

The CB1 receptor was first determined and characterized as the receptor protein for Δ^9^-THC, the major psychoactive constituent in *Cannabis Sativa* (Marijuana), from rat brain preparations in 1988 [1]. Cloning of a CB1 receptor from a rat cerebral cortex [2], followed by the cloning of a human CB1 receptor from the brain stem [3], has prompted ongoing research in the cannabinoid field. The CB1 receptor represents a promising target for the development of novel therapeutics for the treatment of different pathologies, including metabolic syndromes and neurodegenerative diseases, as well as the symptomatic relief of neuropathic pain in patients with multiple sclerosis and spinal cord injuries [4,5]. This broad spectrum of possible therapeutic applications by targeting the CB1 receptor originates from its high expression levels in the central nervous system (CNS), being located primarily at the presynaptic terminals of central and peripheral neurons, and from its neuro-modulatory action [6,7,8]. For example, low expression levels and desensitization of the CB1 receptor have been associated with early (pre-symptomatic) stages of Huntington’s disease (HD) and Parkinson’s disease (PD) [9,10].

Activation of CB1 induces a wide spectrum of intracellular signaling cascades through its coupling to different intracellular effector proteins, including G-proteins and β-arrestins [11]. Each of these signaling cascades results in a unique pharmacological response, which could be exploited to develop novel therapeutics for the treatment of particular disease states. A recent study demonstrated that the endocannabinoids 2-arachidonoylglycerol (2-AG) and anandamide (AEA) are promising candidates for targeting HD, compared with the classical cannabinoids (CP55,940 and ∆^9^-THC) [12,13]. A molecular-level understanding of the structural determinants of biased signaling at the CB1 receptor should contribute to the development of novel therapeutics able to activate diverse signaling paradigms.

## 2. CB1 Signaling

Activation of the CB1 receptor inhibits forskolin-stimulated adenylyl cyclase by coupling to the pertussis toxin (PTX)-sensitive G-protein (Gα_i/o_) and increases the phosphorylation of extracellular signal-regulated kinase 1/2 (pERK1/2) through G-protein dependent and β-arrestin1 dependent pathways [11,14]. The Gα_i/o_ mediated decrease in cAMP upon CB1 activation activates G-protein-coupled inwardly-rectifying potassium channels (GIRKs) and inhibits N-type and P/Q type voltage-gated calcium channels, resulting in an inhibition of pre-synaptic neurotransmitter release. Stimulation of CB1 also leads to phosphorylation and activation of mitogen-activated protein kinases (MAPK), such as p38 MAPK and c-Jun N-terminal kinase, which can regulate nuclear transcription [6,15,16]. While CB1 couples mainly through the Gα_i/o_ type G-protein, coupling to other G-protein types under special circumstances has been also reported, such as coupling to Gα_s_ [17,18,19,20,21], and Gα_q/11_ [22].

### Phosphorylation and Subsequent β-Arrestin Recruitment

In the canonical GPCR signaling pathway, G-protein-coupled receptor kinases (GRKs) phosphorylate serine and/or threonine residues on the intracellular (IC) domain of agonist-activated receptors, mainly at the C-terminus and/or the third IC loop [23]. It has also been reported that individual GRKs elicit distinct phosphorylation patterns (barcodes), resulting in different downstream signaling [24]. β-arrestin recruitment to the phosphorylated receptor depends on the phosphorylation pattern and redirects the G-protein-dependent signaling state of the receptor to other possible states, depending on the type of β-arrestin being recruited. The two non-visual arrestins—β-arrestin1 and β-arrestin2—are expressed ubiquitously in mammalian tissues and mediate GPCR desensitization, endocytosis, ubiquitination, or G-protein independent signaling [25,26]. β-arrestin recruitment to the phosphorylated receptor may sterically inhibit G-protein coupling to the activated receptor, thus quenching the G-protein signal and reducing the receptor’s response to repeated stimulation, known as receptor desensitization [27,28,29]. On the other hand, β-arrestins may act as scaffolding proteins, in which they can mediate clathrin-mediated receptor endocytosis by binding to β(2)-adaptin and clathrin, or they can mediate G-protein independent intracellular signaling pathways by scaffolding mitogen-activated-protein-kinase (MAPK) signaling modules, including the ERK1/2, p38MAPK, and c-Jun N-terminal kinases, as well as scaffolding the Src family tyrosine kinases and protein phosphatases [23,26,30,31,32,33]. In addition, non-canonical G-protein independent β-arrestin recruitment to GPCRs has been reported [25,34,35]. GRK5 and GRK6 have been reported to be able to phosphorylate inactive receptors; this fact may explain the ability of β-arrestins to recruit GPCRs in a G-protein independent way [36]. For example, Org-27569 has been reported to induce a G-protein-independent β-arresin1-dependent pERK1/2 signal in HEK293 cells expressing a CB1 receptor, where the signal was shown to be PTX-insensitive [11].

In vitro studies on the effects of GRKs and arrestins on CB1 receptor signaling, desensitization, and internalization will be discussed in later sections. On the other hand, in vivo studies using β-arrestin1 and β-arrestin2 knockout mice demonstrated that the β-arrestins regulate cannabinoid sensitivity and activity in an agonist-selective (as well as in a region-specific) manner [28,37,38,39]. For example, repeated administration of ∆^9^-THC to mice resulted in the differential upregulation of GRK2, GRK4, β-arrestin1, and β-arrestin2 in different regions of the CNS, while GRK5 and GRK6 levels in the same study were not affected [40]. On the other hand, deletion of β-arrestin2 attenuated tolerance to ∆^9^-THC mediated antinociception, and reduced ∆^9^-THC-induced desensitization in the cerebellum, caudal periaqueductal gray, and spinal cord; yet, it increased desensitization in the hypothalamus, cortex, globus pallidus, and substantia nigra [37]. In a different study, deletion of β-arrestin2 selectively enhanced the antinociceptive and temperature-depressive efficacy of ∆^9^-THC without affecting the efficacy of other ligands, such as CP55,940, methanandamide, JWH-073, and O-1812 [39]. On the other hand, the deletion of β-arrestin1 had no effect on the efficacy or tolerance development (in tail-flick and rectal temperature assays) of ∆^9^-THC. β-arrestin1 deletion, on the other hand, diminished the effects of CP55,940 in both assays, despite an increase in CP55,940-induced [^35^S]GTPγS binding [38]. This may indicate that the antinociceptive and the temperature-depressive effects of CP55,940 are not G-protein dependent.

## 3. Structural Determinant of G-Protein Coupling versus β-Arrestin Coupling at CB1: Insights from Mutation Studies and Crystal Structures

**Figure 1:**
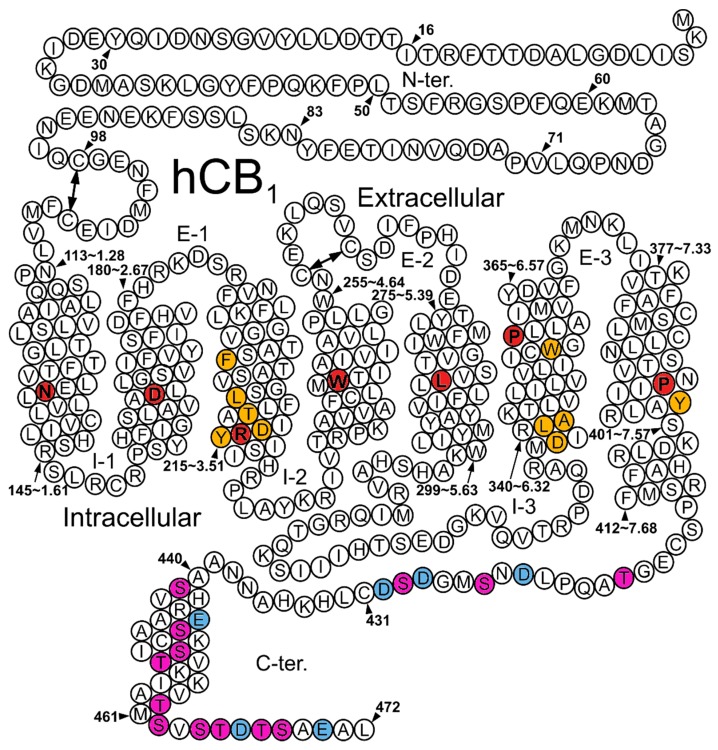
Human CB1 Helix net. (I1–I3 for the intracellular (IC) loops, E1–E3 for the extracellular (EC) loops). The most conserved amino acid residue in each helix in red. Purple highlights for putative phosphorylation sites on the C-terminus of the receptor. Blue highlights for negatively-charged residues in the C-terminus. Other residues discussed are highlighted in orange; in order, they are: F3.36, L3.43, T3.46, Y3.49, D3.51, W6.48, L341(6.33), A342(6.34), D6.30, and Y7.53.

CB1 is a member of the Class A GPCRs and shares their general topological features; seven transmembrane helices (TMH) joined by extracellular (EC) and intracellular (IC) loops of varied lengths, with an extracellularly-extending N terminus, as well as an intracellular C terminus which begins with a short helical segment (Hx8), oriented parallel to the cell membrane. The binding site for endogenous ligands is generally formed by the EC core within the TMH bundle and may extend to the EC loops (referred to as the orthosteric binding site) (Figure 1). Similar to other Class A GPCRs, which are activated by lipid-derived endogenous ligands [41,42,43], the crystal structures of CB1 suggest a transmembrane portal through which an antagonist may diffuse from the lipid bilayer towards the binding site [44,45]. Ligands may also bind to distinct (allosteric) binding sites of the receptor [46,47,48].

Differential coupling of the receptor to G-proteins or β-arrestins in a biased or un-biased fashion is determined by the conformation of the receptor at the IC domain (including the cytoplasmic transmembrane domain, the IC loops, and the C-terminus). Binding of an agonist to the receptor induces a set of ligand-specific conformational changes in the binding site, which are translated into distinct conformational changes in the IC domain. Such changes confer a best fitting between the receptor and specific IC effector protein(s) and result in unique intracellular signals and pharmacological responses. In general, the activation of class A GPCRs results in an opening at the IC domain. Available active state crystal structures reveal a set of concerted rearrangements in conserved motifs within this class of receptors, resulting in an opening at the TMH 3/5/6 region allowing the G-protein C-terminal α5 helical domain to form interactions with the receptor [49,50,51,52,53,54,55]. However, few features are yet known that describe the conformational changes associated with G-protein-independent β-arrestin coupling to the receptor. The published crystal structure of the 5-HT_2B_ receptor bound to β-arrestin biased agonist (LSD), shows a unique rotameric state of conserved tyrosine in TMH7 (Y7.53) adopting a trans χ1 dihedral, compared to the g+ χ1 dihedral commonly seen in the active state crystal structures of GPCRs bound to non-selective agonists [56]. Biophysical studies on the arginine–vasopressin receptor and β2AR have shown a movement of TMH7 in receptors treated with β-arrestin biased ligands, compared to a movement of TMH6 in receptors treated with G-protein activating ligands [57,58].

In addition to their ability to bind GPCRs in the core-conformation (the finger-loop region of arrestins interact with the cytoplasmic core of the receptor), arrestins have also been reported to bind to the receptor in the tail-conformation; where the arrestin is bound to the C-terminus of the receptor only [59]. By interacting in the tail conformation, arrestins preserve their ability to mediate G-protein independent signaling and receptor internalization, but not desensitization [59]. Interestingly, GPCR–G-protein–β-arrestin megaplexes have been reported, in which arrestin is engaged to the GPCR in the tail conformation, allowing concomitant binding of G-protein to the receptor, these megaplexes explain the ability of some GPCRs, including CB1, to activate G-proteins from internalized compartments [60,61,62].

At CB1, an in-situ reconstitution technique was used to directly measure G-protein activation [63], and a Plasmon Wave-guide Resonance (PWR) spectroscopy study [64] demonstrated that different agonists induce different conformational changes in CB1, resulting in the preferential activation of different G-proteins. Fay and Farrens demonstrated differences in the structure of CB1 stabilized by ORG27569 (a biased β-arrestin1 allosteric ligand [65]) from that stabilized by CP55,940 (a non-biased orthosteric agonist [11,17]); specifically, TMH6 movement was blocked, while TMH7/Hx8 movement was enhanced, upon ORG27569 binding [66]. In addition, a recent study demonstrated the different kinetics of Ca^2+^ mobilization after treatment with WIN55,212-2 or CP55,940, suggestive of ligand-specific binding or activation kinetics [67]. Structural determinants of biased signaling at CB1 will be discussed in following sections; those at the cytoplasmic transmembrane domain of the receptor, and the structural determinant at the C-terminus and IC loops.

### 3.1. Structural Determinant of Biased Signaling in the Cytoplasmic Transmembrane Domain of the Receptor

Despite the tremendous variation in chemical structures of agonists that bind and activate Class A GPCRs, GPCRs share common molecular activation mechanisms. This is due to a set of amino acid residues which are highly conserved at the majority of non-olfactory members (ligand-modulated receptors) of this class [68]. These residues are thought to act as molecular switches that undergo concerted conformational changes upon ligand binding and determine the conformation of the receptor at the IC domain. Conserved amino acid residues include the following motifs: NxLV in TMH1, LAxAD in TMH2, the D/ERY motif in TMH3, FxxCWxP in TMH6, and the NSxxNPxxY motif in TMH7. The role of conserved amino acid residues in Class A GPCR signaling, including the CB1 receptor, have been investigated through mutation. However, their exact role in biased signaling is still not well understood, because most of these mutations were evaluated for their canonical G-protein signaling efficacy only, while their efficacy in β-arrestin signaling is yet emerging in the literature. In addition, the signaling properties of those mutants could also be ligand-specific.

The G-protein coupling active state crystal structure of CB1 shows, in agreement with other Class A GPCRs, a counterclockwise rotation (EC view) of the IC domain of TMH6 and flexing in the TMH6 CWxP hinge region, resulting in the outward movement of the IC end of TMH6; away from TMH3. This movement will break the ionic interaction between R3.50 and D6.30 at the IC ends of TMH3/6 [52,53,54,69]. Flexing in the CWxP motif is produced by ligand binding, which alters the χ1 dihedral angles of the “toggle switch” residues. For CB1, these are F3.36 in TMH3 and a tryptophan residue (W6.48) which is part of the CWxP motif [69,70,71,72]. The F3.36A CB1 mutation resulted in increased basal [^35^S]GTPγS binding of the receptor. The authors concluded that F3.36 stabilizes an inactive state of the receptor by stabilizing W6.48 in its inactive rotameric state (χ1 = g+) [70,72], which was confirmed, subsequently, by the CB1 crystal structures [44,45,69] (see Supplementary Materials cb1-sequence-alignment.xlsx for sequence numbering).

Conformational changes in TMH7 and TMH3 have also been shown to occur during G-protein mediated signaling. In the inactive state, TMH7 is packed against TMH3, which is engaged with a conserved change in the χ2 dihedral of Y7.53 by −60°, facilitating its interaction with R3.50 in its active rotameric state (Figure S1). Interestingly, a site-directed fluorescence labeling (SDFL) study using a minimal cysteine CB1 receptor has shown that CP55,940-induced changes in bimane fluorescence occur faster in the Hx8-attached probe (at L7.60), compared to that attached to TMH6 (at A6.34). This may suggest that the inward movement of TMH7/Hx8 precedes the outward movement of TMH6 in G-protein signaling [66]. In addition, the packing of TMH7 against TMH3 is enhanced by a rotational movement of TMH3 towards TMH2, which relieves a steric clash that would occur between Y7.53 and a highly conserved non-polar amino acid residue (L3.43) upon activation (Figure S2). In agreement with this, a CB1 L3.43A mutant displayed higher constitutive activity, demonstrated by a higher basal-specific GTPγS binding, compared to the WT receptor [20,73].

On the other hand, the CB1 receptor has an atypically polar amino acid residue (T3.46), compared with a conserved non-polar (I/L/M/V) residue in most (95%) Class A GPCRs. This residue is located one turn extracellular to the DRY motif. A T3.46I mutant receptor expressed in HEK293 cells exhibited increased constitutive (GTPγS binding) activity and internalization of the receptor, while the T3.46A mutant exhibited lower constitutive activity and had an increased thermal stability, suggestive of a shift towards the inactive state [73,74,75].

The most conserved residue in TMH3 across Class A GPCRs is R3.50. This residue is part of the E/DRY motif. It stabilizes the inactive state of the receptor by forming an interaction with a (usually acidic) residue on TMH6 (D/E6.30); this interaction is broken upon activation of the receptor (in the G-coupling active state) and R3.50 adopts a unique rotameric state, where it forms a triad interaction with both Y7.53 and Y5.58. Despite forming key interactions in both states, the mutation of this residue results generally in reduced basal- and agonist-induced signaling in different receptors, suggestive of a role of this residue in stabilizing the active state of the receptor and/or being involved in the GPCR/G-protein interaction and activation [76,77]. On the other hand, a non-conserved mutation of E/D3.49, which stabilizes the inactive state of the receptor by forming an ionic interaction with R3.50 in the inactive state, resulted unexpectedly in either an enhancement or inhibition of the constitutive activity of different receptors [76,77]. Gyombolai et al. [78] examined the effects of single, double, and triple mutants of the DRY motif in the CB1 receptor, expressed in CHO cells and treated with 2-AG or WIN55,212-2. An R3.50A CB1 mutant displayed a 20% decrease in its ability to recruit Go-proteins without affecting the basal recruitment to the receptor or the EC_50_ values of 2-AG or WIN55,212-2; it, additionally, displayed enhanced basal β-arrestin2 recruitment. The double mutant AAY, on the other hand, reduced Go protein recruitment, with a complete loss of ability to inhibit forskolin-induced cAMP accumulation, and displayed an increase in both basal and maximum β-arrestin1/2 recruitment, suggestive of its β-arrestin biased signaling properties [78]. A similar mutant of the angiotensin-1A receptor has also been reported to be β-arrestin biased [79]. In contrast, a CB1 DAA double mutant showed impaired recruitment of Go-proteins and impaired ability to inhibit forskolin-stimulated cAMP but resulted in a complete loss of β-arrestin1/2 recruitment. An impairment in recruitment of β-arrestins was also characteristic of the single DRA mutant with increased basal Go-protein recruitment [78]. Data suggested a role of Y3.51 in stabilizing a β-arrestin-coupling active state of the receptor, possibly through stabilization of the IC2 loop of the receptor. It is worth mentioning, however, that the same study reported that, for the β-arrestin-biased double mutant (AAY), the pERK signal was no different from the WT receptor; despite the robust increase in β-arrestin1/2 recruitment [78]. This signals the need to differentiate between the ability of the receptor to recruit any of the intracellular effector proteins versus it ability to activate and signal through those proteins.

The most conserved residue in TMH2 (D2.50) has been also explored in different studies. A neutralization mutant of this residue (D2.50N) has been reported to inhibit receptor internalization in ATt20 and HEK293 cells expressing the mutant, without affecting CP55,940- and WIN55,212-2-induced pERK1/2 signals [29,80]. The mutant blocked CP55,940- and WIN55,212-2-induced potentiation of inwardly-rectifying potassium channel (KIR) currents evaluated in ATt20 cells expressing D2.50N and in oocytes co-expressing D2.50N/GIRK1/4 [80,81]. The same mutation did not affect the agonist-induced inhibition of Ca^2+^ currents [80,82] despite the fact that the modulation of both Ca^2+^ and K^+^ currents are G-protein-dependent. On the other hand, inhibition of forskolin-stimulated cAMP accumulation has been found to be attenuated in HEK293 cells expressing a mutant receptor [83]; yet had been reported as not affected in another study, where D2.50N was expressed in ATt20 cells [80].

### 3.2. Structural Determinants at the C-Terminus and the IC Loops

**Figure 2:**
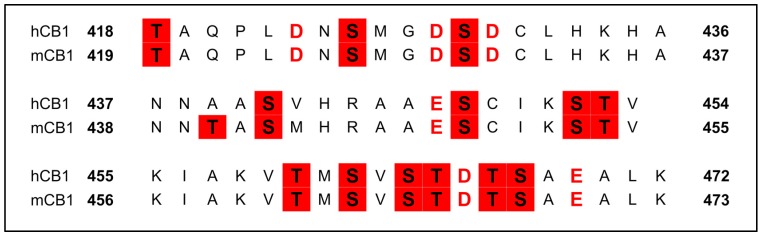
Sequence alignment (hCB1/mCB1) of the C-terminus of CB1. Negatively-charged residues are in red, possible phosphorylation sites are highlighted red.

The C-terminus in CB1 contains multiple serine, threonine, aspartate, and glutamate residues. According to the recently-proposed phosphorylation code, a maximum separation of two amino acid residues between phosphorylated or negatively charged residues is suggested for high-affinity binding to arrestin [84,85]. The membrane-proximal segment of the C-terminus which contains the sequence D423-NSMGDS-D430 (hCB1 numbering), and the membrane distal segment which contains T460-MSVSTDTSA-E470, are two possible β-arrestin interaction sites at the C-terminus of the CB1 receptor. The central segment, on the other hand, contains phosphorylation sites that are separated by more than two amino acid residues, which implies low (or no) interaction of the arrestins with the CB1 receptor at this region (Figure 2).

Crystal structures of the CB1 receptor, in its active and inactive states, resolved only a short segment of the C-terminus, Hx8 [44,45,69]. Efforts to determine the structure of the complete C-terminus revealed a possible variation in the structure upon phosphorylation; an NMR structure of the un-phosphorylated full-length C-terminus (R400–L472) in dodecylphosphocholine revealed an additional amphipathic helical structure (A440–M461) named H9 [86]. On the other hand, two helical segments, extending from L423 to L433 and separated by a glycine residue (G428), were observed for the diphosphorylated peptide segment (T419–N439, phosphorylated at S426/S430) upon binding to β-arrestin1 [87]. In addition, a solution NMR experiment for a pentaphosphorylated peptide segment (T454–L473 phosphorylated at T454, S463, S465, T468, S469) in a complex with β-arrestin1 revealed a helical segment at D467–E471, with no signs of interaction between the un-phosphorylated segment and β-arrestin1 (determined using isothermal titration calorimetry) [88].

#### 3.2.1. G-Protein Interaction with the C-Terminus and IC loops

Different studies demonstrated the involvement of proximal C-terminus (R401–E417), and both the second and third IC loops, in the binding of G-proteins to CB1 [89,90,91,92,93]. Truncation of the distal C-terminus (CB1–417) increases the constitutive activity and G-protein sequestration of the receptor [82], which might be attributed to reduced β-arrestin recruitment to the receptor. An inversion mutant at the third IC loop (L341A–A342L mutation) attenuates the association with Gi-proteins, which has been attributed to a loss of helicity in that loop [94]; this mutant resulted in an increase in basal- and agonist-induced cAMP and has been proposed to couple to Gα_s_ [21,95]. However, the increase in cAMP upon CB1 stimulation has been attributed, in a later study at Howlett’s Lab, to reduced Gα_i/o_ function [95].

#### 3.2.2. β-Arrestin2 Recruitment to Phosphorylated C-Terminus Proximal to the Transmembrane Domain Results in Receptor Desensitization

As discussed earlier, receptor desensitization results from β-arrestin binding to the core of the receptor, which will sterically inhibit the binding of G-proteins to the receptor. The following studies confirm the ability of β-arrestin2 to desensitize the receptor when bound to the phosphorylated C-terminus proximal to the transmembrane domain of CB1.

Mackie’s lab demonstrated the role of GRK3 and β-arrestin2 in CB1 receptor desensitization using *Xenopus* oocytes transfected with rat CB1. Attenuation of G-protein dependent Kir current, during an 8 min exposure to WIN55,212-2, required co-expression of both GRK3 and β-arrestin2. In the same study, truncation mutants of the whole C-terminus (∆418), or (∆418–439) did not result in receptor desensitization, while truncation at 439 and at 460 resulted in WIN55,212-2-induced receptor desensitization. In addition, S426A or S430A mutations significantly attenuated desensitization, while they had no effect on the internalization of the receptor evaluated in AtT20 cells treated with WIN55,212-2 [27]. A subsequent study in HEK293 cells, on the other hand, showed that the S426A/S430A mutant recruits β-arrestin2 to a similar extent in the WT receptor, suggesting the ability of β-arrestin2 to be recruited to the distal C-terminus of the receptor [29]. It is worth-mentioning, here, that β-arrestin2 mRNA has been reported to be expressed in both HEK293 and AtT20 cell lines, while β-arrestin1 mRNA was only expressed in HEK293 cells [96]. Moreover, cultured autaptic hippocampal neurons from mutated (S426A/S430A) mice showed enhanced WIN55,212-2-mediated depolarization-induced suppression of excitation (DSE) and reduced agonist-mediated desensitization of DSE, which has been translated, in vivo, into mutant mice that were more sensitive to the antinociception and hypothermic effects induced by ∆^9^-THC and endocannabinoids, delayed tolerance, and increased ∆^9^-THC-dependence [97]. In a different study, treatment with WIN55,212-2 resulted in desensitization of the ∆419–460 rCB1 receptor transfected into CB1 knockout autaptic hippocampal neurons; the distal segment of the C-terminus (including putative phosphorylation sites at T461–S469) was, thus, directly attached to the transmembrane bundle [98].

An immunoprecipitation study from Howlett’s lab, using cultured N18TG2 neuroblastoma cells, demonstrated that a synthetic phosphorylated proximal C-terminal peptide segment (T419–H436 phosphorylated at S426/S430) and both phosphorylated and non-phosphorylated distal C-terminal peptide segments (V460–L473 phosphorylation at T468) competed for the association of β-arrestin2 with the CB1 receptor [99].

**Figure 3:**
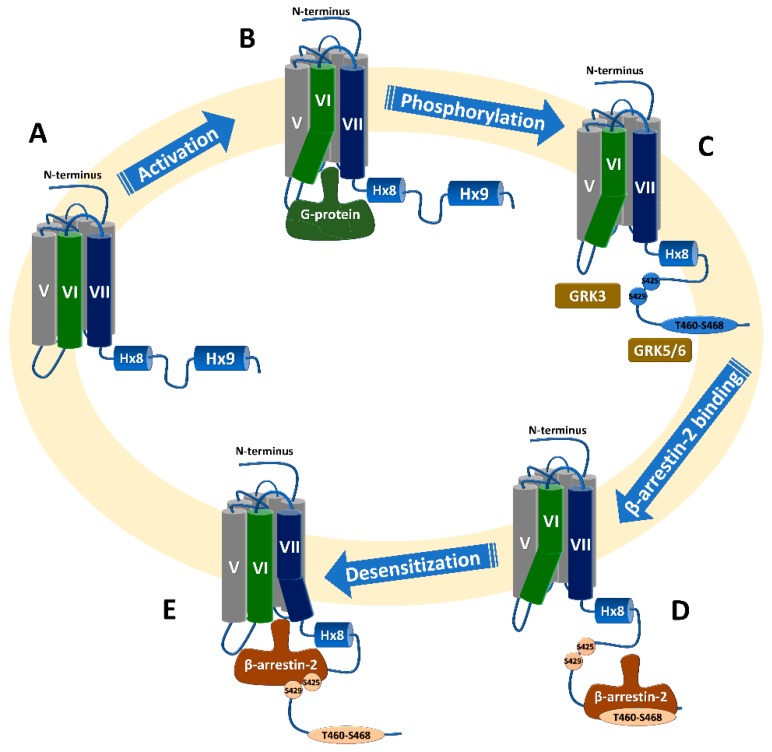
Canonical CB1 receptor signaling and β-arrestin2 interaction with the receptor. (**A**) Inactive state of the receptor with closed IC domain. (**B**) Agonist binding activates the receptor with opening at IC domain, mainly due to the TMH6 conformational change followed by G-protein coupling. (**C**) Phosphorylation of proximal (S425, S429) and distal (possibly at T460–S468) phosphorylation sites, by GRK3 and GRKs5/6, respectively. (**D**) β-arrestin2 binding to the C-terminus starts with an interaction with the distal phosphorylated C-terminus, which can mediate receptor internalization; followed by (**E**) possible conformational change at the IC domain of the receptor and the arrestin interacts with the proximal phosphorylated C-terminus, resulting in receptor desensitization.

Results from all the above studies suggest that β-arrestin2 can be recruited to the phosphorylated proximal C-terminus in the WT receptor, resulting in receptor desensitization, while it can still be recruited to the distal region of the C-terminus in the absence of phosphorylation at S426/S430, resulting in receptor internalization but not desensitization, as will be discussed below (Figure 3).

#### 3.2.3. β-Arrestin2 Recruitment to Phosphorylated C-Terminus Distal to the Transmembrane Domain Results in Receptor Internalization

While the phosphorylation pattern at the proximal C-terminus seems to be important for receptor desensitization, phosphorylation at the distal C-terminus has been proposed to be implicated in receptor internalization [100]. β-arrestin2 has been determined to be essential for agonist-induced internalization of the receptor [65,101].

To determine critical phosphorylation site residues required for internalization, Mackie’s lab introduced different mutations to the distal C-terminus of the CB1 receptor, expressed in HEK293 cells. T461A/S463A, S465A/T466A, and T468A/S469A double mutants showed a similar extent of internalization and an efficient, but slower, rate of β-arrestin2 recruitment to the plasma membrane compared to the WT, upon treatment with CP55,940. However, mutating four or all six putative phosphorylation sites (T461A–T466A and T461A–S469A) significantly attenuated CP55,940-induced receptor internalization, and did not show translocation of β-arrestin2 to the membrane [100].

Interestingly, the same study reported that a CB1 receptor lacking the last 14 amino acid residues (V460Z) and expressed in HEK293 cells internalized to the same extent as the WT receptor and could recruit β-arrestin2 to the membrane [100]. A possible scenario of the interaction of β-arrestin2 with the C-terminus of the receptor could be proposed, based on those results; β-arrestin2 binding to the C-terminus of the WT receptor is initiated with an interaction with the distal phosphorylated C-terminus, followed by interaction with the proximal phosphorylated C-terminus region of the receptor. It can also suggest that the distal C-terminus has a specific (and not random) location, with respect to the proximal C-terminus, and that the binding of β-arrestin2 to the proximal C-terminus can also induce receptor internalization. Thus, β-arrestin2 could recruit to double mutants (T461A/S463A, S465A/T466A, and T468A/S469A) inducing internalization due to the availability of phosphorylation sites at the distal C-terminus; but, it could not recruit to the receptor with four and six mutations (T461A–T466A and T461A–S469A) due to lack of phosphorylation sites at those residues and because the non-phosphorylated distal C-terminus occludes other phosphorylation sites at the proximal C-terminus. However, truncation of the distal C-terminus (V460Z) permitted direct interaction of β-arrestin2 with the proximal phosphorylated C-terminus, which resulted in receptor internalization (see Figure 3).

#### 3.2.4. β-Arrestin1 Recruitment to the Receptor Results in G-Protein Independent Signal

Phosphorylated proximal and distal peptide segments have been shown to bind β-arrestin1, as discussed earlier (see Section 3.2). However, an immunoprecipitation study from Howlett’s lab, using cultured N18TG2 neuroblastoma cells, suggested that β-arrestin1 interacts only with the proximal C-terminus (T419–H436) phosphorylated at S426/S430, and with the distal C-terminus (V460–L473) phosphorylated at T468. They also reported the inability of a non-phosphorylated proximal peptide segment to compete with β-arrestin1 for the association with CB1 [102]. On the other hand, a recent study demonstrated significantly higher recruitment of β-arrestin1 to CB1 S426A/S430A, compared to the WT receptor expressed in HEK293 cells. The same study found a weaker recruitment of β-arrestin2 to the CB1 S426A/S430A mutant, compared to the WT receptor 5 min after treatment with WIN55,212-2. The mutant also resulted in an interesting shift in the functional selectivity of agonists (WIN55,212-2 and 2AG) towards inducing a biased β-arrestin1-dependent pERK1/2 signal in the mutant receptor [14]. Such paradoxical results from different labs could be due to the expression of CRIP1a in N18TG2 cells, but not in HEK293 cells [103]. CRIP1a competes with arrestins for binding to a non-phosphorylated C-terminus, which explains the inability of the non-phosphorylated proximal peptide segment to recruit β-arrestin1 for its association with CB1 in N18TG2 cells [102]. A G-protein-independent β-arrestin1 pERK1/2 signal has also been reported for ORG27569, as will be discussed in Section 4.3.2.

#### 3.2.5. Recruitment of β-Arrestins to the C-Terminus Can be Altered in the Presence of Other Regulatory Proteins

Expression of regulatory proteins that bind to the C-terminus of the CB1 receptor may alter agonist-dependent/independent arrestin recruitment to the CB1 receptor. The cannabinoid receptor interacting protein 1a (CRIP1a) has been demonstrated to interact mainly with non-phosphorylated C-terminus of the CB1 receptor [102]. CRIP1a is a 164 amino acid residue protein with a predicted palmitoylation site but no transmembrane domain, which has high expression in certain brain regions, including the cerebral cortex, cerebellum, hippocampus, hypothalamus, and caudate nucleus. In vivo co-expression has been determined using a co-immunoprecipitation technique from rat brain homogenates [103]. Furthermore, CRIP1a colocalization with the CB1 receptor at presynaptic termini was also confirmed, using immune-histochemical studies in transgenic mice cerebellum [104]. CRIP1a has been reported to attenuate agonist-induced CB1 receptor internalization [104], and modulate CB1 mediated activation of G-proteins in a subtype selective manner [102]. CRIP1a binds preferentially to the non-phosphorylated C-terminus [99]. Its competition with arrestins for binding to the CB1 C-terminus has been proposed to explain the inability of a truncation mutant (V460Z), expressed in AtT20 cells, to internalize, despite its ability to internalize in HEK2093 cells [100,105]. Lack of β-arrestin1 expression in AtT20 cells should also be considered when comparing results from HEK293 cells [96]. In addition, V460Z or CB1 T461A–S469A transfected into CB1 knockout autaptic hippocampal neurons did not desensitize following WIN55,212-2 or 2-AG treatment, despite the availability of proximal phosphorylation sites in the mutated receptors [98].

In addition to CRIP1a, Src homology 3-domain growth factor receptor-bound 2-like (endophilin) interacting protein 1 (SGIP1) is an 828 amino acid residue protein with a N-terminal lipid-binding domain. SGIP1 has been shown to interact with the CB1 receptor C-terminus and to inhibit clathrin-mediated endocytosis of the CB1 receptor. It has also been demonstrated to enhance CB1 receptor association with β-arrestin2, but not with β-arrestin1, and to lower WIN55,212-2-induced PTX-sensitive ERK1/2 phosphorylation, while not affecting CB1 receptor mediated Gα_i/o/q_ activation [106]. In addition, SGIP1 has been demonstrated to co-localize at presynaptic termini with the CB1 receptor in cultured cortical neurons, which may explain neuronal compartment-selective endocytosis in which rapid CB1 receptor internalization is observed in neuronal soma, while resistance to endocytosis is observed in presynaptic termini [32,106].

#### 3.2.6. Phosphorylation of the C-Terminus

As discussed earlier, co-expression of GRK3 and β-arrestin2 in oocytes has been reported to be essential for WT CB1 receptor desensitization [27]. Inhibition of GRK2 in N18TG2 cells did not significantly affect CP55,940-induced internalization of the CB1 receptor [99]. In another study, knocking down of GRK3 or GRK2/3 in HEK293 cells expressing rat CB1 receptors significantly increased the pERK1/2 signal 15 min after WIN55,212-2 treatment, while knocking down GRK3 or GRK2/3 had no effect on a CB1 S426A/S430A mutant receptor pERK1/2 signal [14]. As the 15 min WIN55,212-2-induced pERK1/2 signal was determined to be G-protein dependent in the WT receptor [14], it can be concluded that knocking down GRK3 (which phosphorylates proximal phosphorylation sites—specifically at S426 and S430—in the C-terminus) reduces desensitization and internalization of the receptor, resulting in a sustained G-protein dependent pERK1/2 signal in the WT receptor.

On the other hand, GRK4,, GRK5, or GRK6 knock-down resulted in a significantly lower pERK1/2 signal in a CB1 S426A/S430A mutant, but not in the WT receptor after treatment with WIN55,212-2 [14]. The WIN55,212-2-induced pERK1/2 signal has been determined to be β-arrestin1-dependent in the S426A/S430A mutant receptor [14], and β-arrestin1 mediated signaling through the CB1 receptor has been reported, previously, to be from endocytic pits [11]. If GRK4, GRK5, and GRK6 were involved in the phosphorylation of distal phosphorylation sites which are essential for receptor internalization, knocking down those GRKs may have reduced the internalization of the mutant receptor into endocytic pits, resulting in a lower β-arrestin1 mediated pERK1/2 signal in the S426A/S430A mutant. In cultured smooth muscle cells, GRK5 (but not GRK2) has also been reported to be essential for the phosphorylation of CB1 after a 15 min treatment with 1 μM AEA, resulting in CB1 receptor internalization and PTX-insensitive β-arrestin1/2-dependent ERK1/2 and Src kinase activation [107].

Other than GRKs, the CB1 receptor IC3 loop has been reported to be phosphorylated at S317 by PKC in AtT20 cells transfected with rat CB1 receptor. Phosphorylation at S317 has been shown to attenuate CB1 mediated activation of K_ir_ currents [108].

## 4. CB1 Biased Ligands

The pharmacology of numerous CB1 orthosteric and allosteric ligands has been deeply investigated in different in vitro and in vivo models [109,110]. Research into their possible functional selectivity has increased recently, due to the emergence of biased signaling as a novel potential therapeutic approach. CB1 ligands of endogenous, phytogenic, and synthetic nature have been reported to elicit biased cellular responses, as observed by using diverse functional endpoints. In this section, we will summarize and classify the CB1 biased ligands identified so far, attending to their chemical structure.

### 4.1. Endocannabinoids

**Figure 4:**
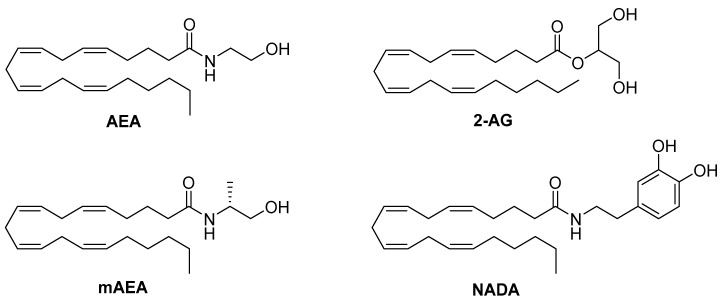
Structures of the CB1 functionally-selective endocannabinoids AEA, 2-AG, mAEA, and NADA.

Diverse endogenous cannabinoids, including the well-known lipids 2-arachidonoylglycerol (2-AG) and *N*-arachidonoylethanolamine (ananadamide, AEA) (Figure 4), have been reported to exhibit a CB1 biased signaling profile in specific cellular models. For instance, as described by Laprairie et al., 2-AG and AEA displayed a functional selectivity in in vitro models of medium spiny projection neurons expressing wild-type (ST*Hdh*Q7/Q7) or mutant huntingtin protein (ST*Hdh*Q111/Q111) and using the Black and Leff operational model to quantify signaling bias [13,17]. According to these authors, 2-AG elicited signaling bias toward Gα_i/o_, compared to β-arrestin1 and Gα_q_ [17], while another report showed that, in rat hippocampal neurons, 2-AG mediates autaptic long-term potentiation through activation of CB1 Gα_q_ [111]. These results indicate that the biased signaling of 2-AG at CB1 might be cell-type dependent. Additional investigations exhibited that this endogenous CB1 agonist is more efficacious at recruiting β-arrestin1 than β-arrestin2 in CB1-HEK293 cells [14]. These authors observed that 2-AG mediated CB1 downstream signaling by G-proteins up to 5 min after incubation, after which it switched to β-arrestin-mediated signaling mechanisms.

It is also worth mentioning that, in a study carried out by Khajehali et al., in which they investigated the effects of CB1 ligands in cAMP inhibition and pERK1/2 assays in CHO-CB1 cells, 2-AG did not show a specific preference for any of those signaling outcomes [35].

Similar to 2-AG, Laprairie et al. reported that, in ST*Hdh* cells, AEA signaling was biased toward Gβγ, compared to Gα_i/o_, and Gα_i/o_-biased, compared to β-arrestin1 and Gα_q_ [13,17]. However, in contrast to 2-AG, AEA exhibited a clear preference toward cAMP inhibition, compared to pERK1/2, in the previously-mentioned experiments carried out by Khajehali et al. [35].

The synthetic chiral analog of AEA, (R-(+)-methanandamide, mAEA, Figure 4), displayed functional selectivity in a study performed in N18TG2 cells. This compound exerted agonism through Gα_i3_, while being an inverse agonist at Gα_i1_ and Gα_i2_ [112]. As previously observed for AEA, mAEA exhibits a preference toward cAMP inhibition, compared to pERK1/2 [35].

More recently, the endocannabinoid and endovanilloid ligand, *N*-arachidonoyldopamine (NADA, Figure 4) has been reported to exhibit biased agonism at CB1 [113]. Redmond and colleagues demonstrated that this ligand exerts a unique CB1 signaling profile, showing significant bias in activating Gα_q_-mediated responses. This effect seems to occur at high concentrations and in a cell- and tissue-dependent manner.

**Figure 5:**
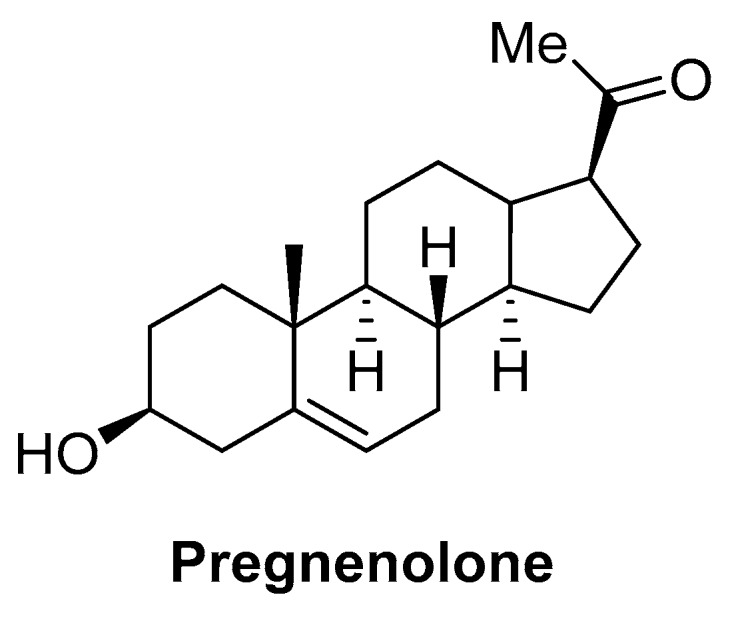
Structure of the CB1 endogenous allosteric modulator pregnenolone.

**Figure 6:**
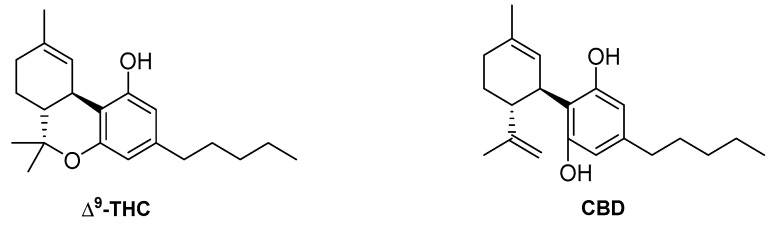
Structures of the plant-derived ligands (−)Δ^9^-tetrahydrocannabinol (Δ^9^-THC) and cannabidiol (CBD).

Besides the aforementioned eicosanoids, a totally different endogenous chemical structure has also been reported to act at CB1 in a biased way. This is the case of the steroid hormone pregnenolone (Figure 5), which acts as a CB1 negative allosteric modulator of Δ^9^-tetrahydrocannabinol (Δ^9^-THC, Figure 6). This endogenous molecule has been reported to elicit biased responses, inhibiting pERK1/2 signaling while not modifying cAMP signaling [48].

The data collected thus far is not enough to determine the molecular basis of CB1 activation of specific signaling pathways by endocannabinoids. Therefore, further studies extending structural diversity among these lipids are clearly needed. The use of a broader spectrum of functional assays and cellular types may also help in understanding each ligand’s pharmacological profile under different pathophysiological conditions.

### 4.2. Phytocannabinoids

Thus far, very few phytogenic cannabinoids have been reported to elicit biased signaling responses. The major bioactive constituent in *Cannabis Sativa*, (−)Δ^9^-tetrahydrocannabinol (Δ^9^-THC), has been described to be biased toward β-arrestin1 recruitment at CB1. Laprairie et al. demonstrated that, in ST*Hdh* cells, Δ^9^-THC exhibits signaling biases toward β-arrestin1, Gα_q_, and Gβ_γ_, compared to Gα_i/o_ [17]. More recently, these authors demonstrated that this compound is able to preferentially enhance the recruitment of β-arrestin1 and reduce cellular viability in an in vitro model of Huntington disease [13].

Previous studies, conducted by Breivogel et al., reported the sensitivity of this psychoactive phytocannabinoid to β-arrestin2 in vivo. Δ^9^-THC was able to produce better antinociception and greater body temperature decreases in β-arrestin2 knock-out mice, compared to their homozygous wild-type counterparts [28,39].

The non-psychotropic cannabinoid cannabidiol (CBD, Figure 6), has also been explored to characterize its possible functional selectivity at CB1. The cannabinoid pharmacology of CBD is still quite intriguing [114]. Different reports showed that it displays a low affinity for CB1 [115], while in vitro studies demonstrated its CB1 antagonistic properties [116,117]. More recently, Laprairie et al. reported that CBD behaves as a CB1 negative allosteric modulator (NAM) of Δ^9^-THC and 2-AG [118]. In this context, two different investigations evaluated the potential CBD signaling bias at CB1. On one hand, studies in ST*Hdh* cells showed that CBD administration only evoked significant Gα_s_-mediated pCREB and, therefore, bias values could not be considered for this ligand. However, the combination of Δ^9^-THC and CBD induced signaling biased toward Gα_s_, compared with Gα_i/o_, while being biased toward Gα_i/o_, compared with β-arrestin1, Gα_q_, and Gβ_γ_ [13,17]. On the other hand, Navarro et al. assessed CBD´s effects by using four different functional outcomes and observed that this phytocannabinoid induces changes in the response of CB1 to different agonists in a biased manner [119]. Moreover, they showed that CBD can also alter the signaling of Δ^9^-THC acting on CB1-CB2 heteromers.

The potential functional bias of a wider range of phytocannabinoids, including their acidic metabolites, should be further explored in diverse cellular systems. This will aid in developing an understanding of the structural relevance of the aliphatic moiety, the phenolic core, or the cyclohexenyl ring for each pathway, guiding future drug design.

### 4.3. Synthetic Cannabinoids

Certain synthetic cannabinergic drugs have been identified as biased ligands at CB1. Even if not many scaffolds have been discovered to exhibit functional selectivity at this receptor, the knowledge gained recently has contributed to progress in this field.

#### 4.3.1. Phytocannabinoid Synthetic Derivatives.

**Figure 7:**
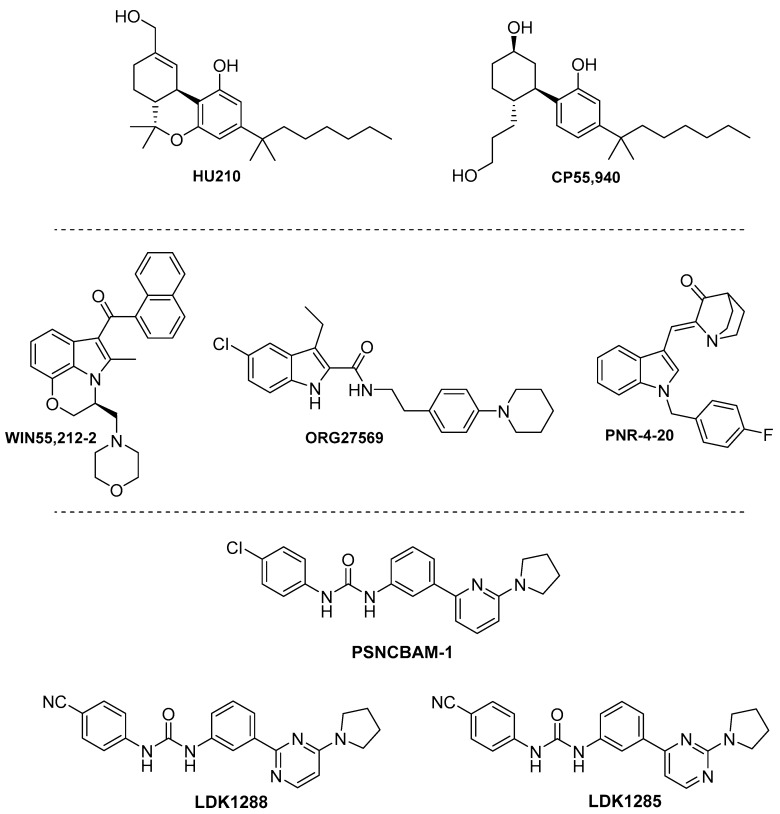
Structures of the phytocannabinoid synthetic ligands HU210 and CP55,940; indole derivatives WIN55,212-2, ORG27569, and PNR-4-20; and biphenylureas PSNCBAM-1, LDJ1288, and LDK1285.

Synthetic cannabinoids, such as CP55,940 and HU210 (Figure 7), have been explored through diverse functional outcomes in observing specific signaling trends. The (–)-1,1-dimethylheptyl analog of 11-hydroxy-Δ^8^-tetrahydrocannabinol, HU210, was shown to elicit a significant bias towards cAMP inhibition (Gα_i/o_ stimulation) over pERK1/2 activation [35]. This compound elicited similar maximal stimulation of Gα_o_ and Gα_i_ in Sf9 cells [63] and displayed equipotent Gα_i_ versus Gα_s_ signaling [120,121]. Moreover, Lauckner et al. showed that, in CB1-HEK293 cells, this compound did not increase calcium release through a Gα_q_ pathway, thus preferring Gα_i_ signal transduction [22].

The widely-used CB1/CB2 agonist CP55,940 [122] also exhibited functional selectivity under specific cellular conditions. Like HU210, CP55940 exhibited a preference for cAMP inhibition, compared to pERK1/2, in CB1-HEK293 cells [35]. However, in contrast to the previous synthetic cannabinoid, CP55,940 favored coupling to Gα_i_ versus Gα_s_ [120,121]. Moreover, Laprairie and coworkers demonstrated that, in ST*Hdh* cells, CP55,940 induced signaling biased toward Gα_s_ and β-arrestin1 compared to Gα_i/o_ [17].

Despite these findings, CP55,940 is usually considered to be an unbiased CB1 full agonist [123]. Divergences among assays may originate from intrinsic properties of the receptor, as well as the functional outcome or cell type/tissue used for the evaluation.

#### 4.3.2. Indoles

Diverse indole chemotypes, including the aminoalkylindole WIN55,212-2, the indole-2-carboxamide ORG27569, and the indole quinulidinone PNR-4-20, have been reported to signal through CB1 in a biased manner.

The potent cannabinoid agonist WIN55,212-2 has been widely investigated using diverse functional outcomes and exploring different signaling pathways. For instance, Glass and Northup observed that, in membranes of CB1 expressing Sf9 cells, WIN55,212-2 elicited maximal Gα_i_ activation, whereas it only partially stimulated Gα_o_ [63]. Studies from Lauckner et al. revealed that, at high concentrations, this aminoalkylindole increased the release of intracellular calcium in CB1-HEK293 cells through stimulation of the Gα_q_ pathway. Therefore, these authors suggested that WIN55,212-2 is able to stabilize CB1 receptors into a conformation that enables Gα_q_ signaling, consequently shifting the G-protein specificity [22]. Moreover, it was reported that WIN55,212-2 exerts equipotent Gα_i_ and Gα_s_ signaling [120,124]. It is also interesting to remark that co-immunoprecipitation studies of activated G-proteins in N18TG2 cells demonstrated that WIN55,212-2 behaved as a full agonist for the Gα_i_ subtypes Gα_1_, Gα_2_, and Gα_3_ [112]. In agreement with previous studies, Laprairie et al. observed that it activates Gα_q_, Gα_i_ and G_βγ_ [17]. Furthermore, the arrestin pathway has been extensively investigated by Delgado-Peraza et al. [14]. These authors demonstrated that this compound was able to induce prolonged activation of pERK1/2 in the mutant receptor (described in previous sections) which is exclusively mediated by β-arrestin1. WIN55,212-2 was able to recruit β-arrestin1 more efficiently than β-arrestin2, being therefore biased towards β-arrestin1.

In 2005, the indole-2-carboxamide ORG27569 was described as the first CB1 allosteric modulator [75,125]. It behaves as a positive allosteric enhancer of agonist binding; however, it is functionally considered a negative allosteric modulator (NAM). Additional studies exhibited controversial pharmacological data for ORG27569, revealing that the allosteric effects of ORG27569 at CB1 might be pathway-specific and time-dependent [35,126]. These results suggest a more complex process than the allosteric profile initially proposed.

In this context, experiments in CB1-HEK293 cells revealed that ORG27569 produces an increase in phosphorylation of ERK1/2 that is not abrogated upon PTX treatment. This study indicates that the observed signaling is not G-protein mediated [127]. Moreover, the role of β-arrestins in G-protein-independent ORG27569 signaling was assessed using siRNA co-transfection, demonstrating that β-arrestin1 mediates the ERK1/2 phosphorylation stimulated by this molecule [127]. However, inverse agonism of ERK signaling by ORG27569 has also been reported [128].

This indole-2-carboxamide was the first biased allosteric modulator described [35,127,129], opening new avenues for the development of subtype- and pathway-selective CB1 therapeutics. Despite the promising signaling mechanism of these types of ligands, their pharmacological profiling is especially challenging and further in vivo assays are needed to demonstrate the therapeutic outcome of the complex biased signaling produced by ORG27569 at the CB1 receptor.

Prather et al. have recently reported the CB1 ligand bias profile of specific indole quinulidinones [123]. This chemotype was previously identified by the same authors, who developed several potent and efficacious CB1 agonists [130,131]. The indole quinulidinone PNR-4-20 (Figure 7) was shown to elicit robust G-protein-dependent signaling, with transduction ratios comparable to non-biased CB1 agonists [123]. However, in contrast to non-biased ligands, this compound is not able to recruit β-arrestin2. Due to this reduced arrestin recruitment, upon chronic exposure of cells to PNR-4-20, significantly lower desensitization and down-regulation of CB1 receptors was detected when related to a similar treatment with non-biased molecules. The indole quinulidinone derivative PNR-4-02 (an analogue of PNR-4-20, in which the fluorine is replaced by a chlorine group) exhibited a similar CB1 bias profile, displaying efficacy for coupling CB1 to G-protein-dependent, β-arrestin2-independent signaling pathways [123]. Ligands with this CB1 functionally-selective profile may offer the advantage of producing less severe adverse effects, even upon chronic treatment.

#### 4.3.3. Biphenylureas

The pyrrodinyl biphenylurea scaffold, PSNCBAM1 (Figure 7), was discovered as an allosteric modulator of CB1 by Horswill et al. over a decade ago [132]. Follow-up structural studies allowed for the identification of pharmacophoric moieties leading to the allosteric properties of this chemotype. For instance, substitution of the chloro- group with a cyano- group generated analogues with a similar pharmacological profile [133]. Further studies, by Kendall et al., enabled the development of the pyrimidinyl biphenylureas LDK1288 and LDK1284 (Figure 7) as novel promising lead molecules. Replacement of the pyridine moiety of PSNCBAM1 with a pyrimidine core led to novel CB1 positive allosteric modulators that exhibit a pathway-specific signal transduction [134]. These new compounds positively modulate the binding of the CB1 orthosteric agonist CP55,940 while exhibiting an antagonism of G-protein coupling. Remarkably, the pyrimidinyl biphenylureas demonstrated ERK1/2 phosphorylation, mediated by β-arrestin1 [134,135]. Therefore, these allosteric modulators may help stabilize a CB1 active conformation that binds to β-arrestin1, while precluding G-protein coupling. Due to their unique pharmacological profile, this promising scaffold may provide a novel anti-obesity approach, mediated through a CB1 biased allosteric mechanism.

Despite the discovery of the aforementioned CB1 biased ligands, this field is yet emerging and further studies need to optimize the bias quantification methods. Relative signaling efficacies and especially the presence of scaffolding proteins may differ in diverse tissues and cell types. This fact, along with intrinsic receptor properties, should be consistently studied to be able to reliably classify the signaling bias of CB1 ligands. In addition, the time points selected to measure cellular endpoints should be standardized to equally account for differences in ligand kinetics. In this context, further structure activity relationship studies, over a wide range of cannabinoid scaffolds and using a consistent bias quantification, will help to identify key structural features and may guide future drug design.

It is worth mentioning that CB1 allosteric modulators may influence ligand bias by affecting the conformation of the active state of a given receptor. They could promote an alternative active conformation that prevents G-protein coupling while promoting β-arrestin recruitment, or vice versa. In this context, as previously mentioned, biased allosterism is now emerging at CB1 and should be further explored. In fact, the biased signaling of allosteric modulators may offer unique therapeutic strategies, conferring selective fine-tuning of CB1-impacted pathways and, thus, reducing the undesirable effects associated with orthosteric ligands.

Moreover, ligand bias can be influenced by receptor oligomerization in specific cells or tissues. For instance, CB1-dependent Gα_q_ signaling has been reported to occur through CB1-D2 (type 2 dopamine receptor) dimerization [136].

In addition to the numerous experimental challenges already mentioned, the actual goal of studying CB1 ligand bias is to identify the correlation of specific signaling cascades and effector proteins with behavioral and physiopathological results in vivo. This knowledge will aid the development of therapeutically-optimized strategies, targeting pathways affected by particular conditions or symptoms.

## 5. Conclusions

This article aims to provide a comprehensive perspective of the structural knowledge gained so far in the CB1 biased agonism field. Differential activation at the CB1 receptor may offer fine-tuned therapeutic strategies for targeting specific symptoms or pathologies. Therefore, a molecular understanding of the structural features that trigger and accompany the activation of each independent pathway, in a biased manner, may provide further insights to move CB1 biased ligands towards the clinic.

## Supplementary Materials

Supplementary materials can be found at https://www.mdpi.com/1422-0067/20/8/1837/s1.

## Abbreviations

CB1	Cannabinoid receptor type 1
THC	Tetrahydrocannabinol
CBD	Cannabidiol
pERK1/2	Phosphorylation of extracellular signal-regulated kinase 1/2
AEA	Anandamide
2-AG	2-Arachidonoylglycerol
PWR	Plasmon Wave-guide Resonance
PTX	Pertussis Toxin
HD	Huntington’s Disease
PD	Parkinson’s Disease
MAPK	Mitogen-Activated Protein Kinases
GIRK	G-protein-coupled inwardly rectifying potassium channels
GRK	G-protein-coupled Receptor Kinase
TMH	Transmembrane helix
pERK	Phosphorylated extracellular regulated kinases
GPCR	G protein-coupled receptor
NMR	Nuclear Magnetic Resonance
MS	Mass Spectrometry
FRET	Förster resonance energy transfer
EPR	Electron paramagnetic resonance
