# Supplementary material for: Structural Insights into CB1 Receptor Biased Signaling
Source: Int J Mol Sci. 2019 Apr 13;20(8):1837. doi: 10.3390/ijms20081837 (PMC6515405; doi:10.3390/ijms20081837)
Supplement: Supplementary file 1 [file ijms-20-01837-s001.zip › 1_SupplementaryMaterial1_Figures_.docx]

**Supplementary Material**

Structural Insights into CB1 Receptor Biased Signaling

Rufaida Al-Zoubi^1,#^, Paula Morales^2,#^ and Patricia H. Reggio^3,^*

**Contents**

**Figure S1.** Conserved conformational changes at the IC domain of class A GPCRs upon receptor activation (G-protein coupling state).

**Figure S2.** The role of L3.43 in stabilizing inactive state conformation.


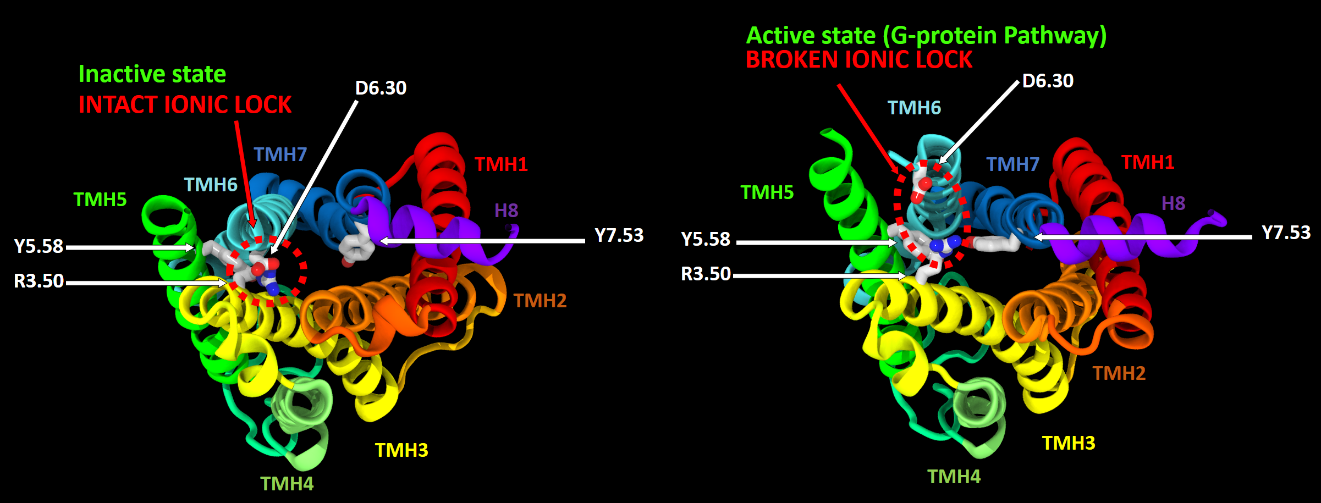


**Figure S1.** **Conserved conformational changes at the IC domain of class A GPCRs upon receptor activation (G-protein coupling state).** An IC view showing transmembrane helices rearrangements, and conformational changes in conserved residues; Y7.53, Y 5.58, and R3.50 (shown as sticks) in crystal structures of the CB1 receptor (PDB IDs: 5U09, 5XRA): comparing the R*G state (Left) vs the R state (Right); activation includes an inward movement of IC domain of TMH7, allowing Y7.53 to reposition between TMH3 and TMH6 stabilizing the outward movement of TMH6 and interacting with R3.50 and Y5.58. This is accompanied by rotamer change of the χ2 and χ3 dihedrals of R3.50 to trans. The χ2 dihedral of Y7.53 is shifted by -60 degrees.


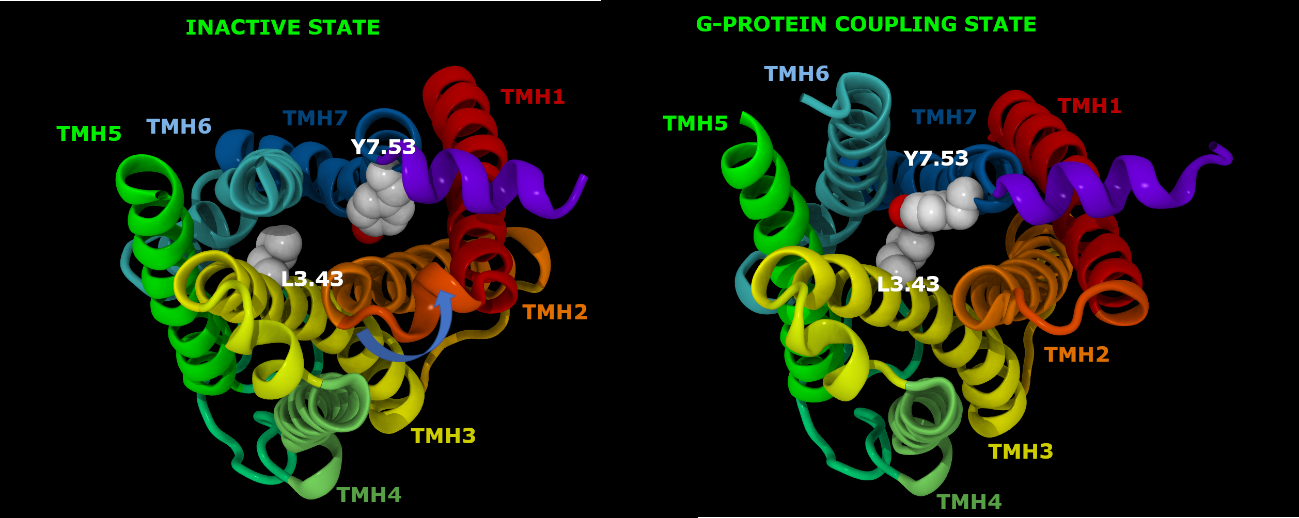

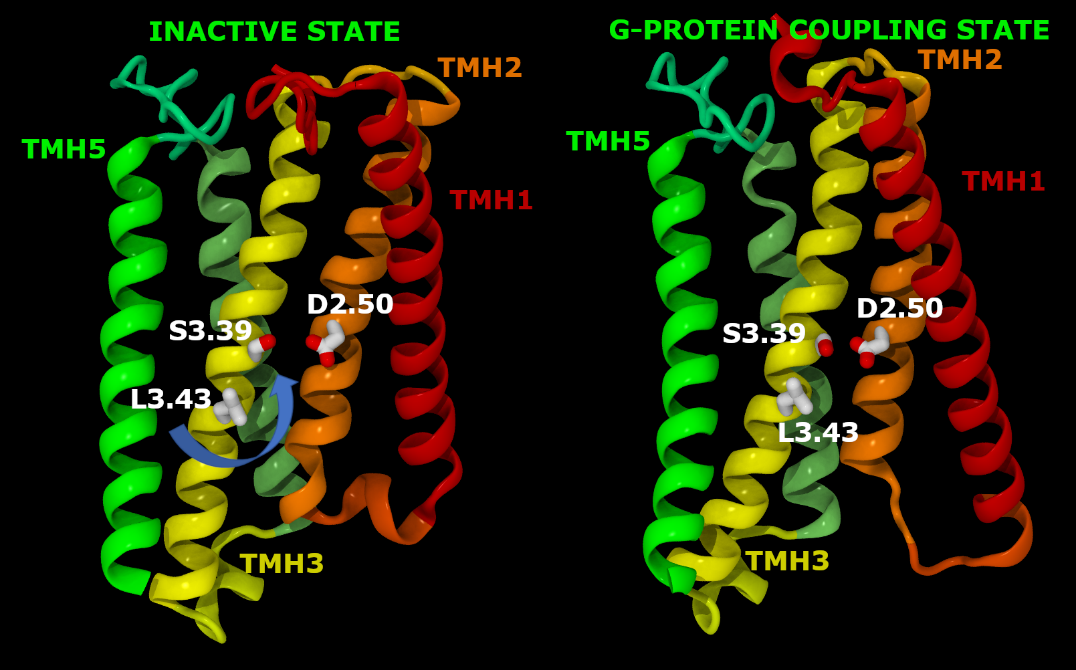


**A**

**B**

**Figure S2.** **The role of L3.43 in stabilizing inactive state conformation.** A) Intracellular view of the inactive (PDB ID: 5U09, Left) and active (PDB ID: 5XRA, Right) states. L3.43 stabilizes the inactive state of the receptor by sterically hindering packing of TMH7 against TMH3 which is required for the transition towards the G protein-coupling active state conformation. B) A side view. (TMH6 and TMH7 were removed for clarity). Rotation of TMH3 towards TMH2 reduces the distance between the Cα atoms of D2.50 and S3.39 by 1.8 Å, and relieves a steric clash that would have occurred between L3.43 and Y7.53 upon activation.
